# Prevalence and morphometric analysis of three-rooted mandibular first molars in a Brazilian subpopulation

**DOI:** 10.1590/1678-775720150511

**Published:** 2016

**Authors:** Clarissa Teles Rodrigues, Christiano de Oliveira-Santos, Norberti Bernardineli, Marco Antonio Hungaro Duarte, Clovis Monteiro Bramante, Paloma Gagliardi Minotti-Bonfante, Ronald Ordinola-Zapata

**Affiliations:** 1 Universidade de São Paulo, Faculdade de Odontologia de Bauru, Departamento de Dentística, Endodontia e Materiais Odontológicos, Bauru, SP, Brasil.; 2 Universidade de São Paulo, Faculdade de Odontologia de Ribeirão Preto, Departamento de Estomatologia, Saúde Coletiva e Odontologia Legal, Ribeirão Preto, SP, Brasil.

**Keywords:** Anatomy, Cone-beam computed tomography, Root canal therapy, X-ray microtomography

## Abstract

The knowledge of the internal anatomy of three-rooted mandibular molars may help clinicians to diagnose and plan the root canal treatment in order to provide adequate therapy when this variation is present.

**Objectives:**

To determine the prevalence of three-rooted mandibular molars in a Brazilian population using cone beam computed tomography (CBCT) and to analyze the anatomy of mandibular first molars with three roots through micro-CT.

**Material and Methods:**

CBCT images of 116 patients were reviewed to determine the prevalence of three-rooted first mandibular molars in a Brazilian subpopulation. Furthermore, with the use of micro-CT, 55 extracted three-rooted mandibular first molars were scanned and reconstructed to assess root length, distance between canal orifices, apical diameter, Vertucci's classification, presence of apical delta, number of foramina and furcations, lateral and accessory canals. The distance between the orifice on the pulp chamber floor and the beginning of the curvature and the angle of canal curvature were analyzed in the distolingual root. Data were compared using the Kruskal-Wallis test (α=0.05).

**Results:**

The prevalence of three-rooted mandibular first molars was of 2.58%. Mesial roots showed complex distribution of the root canal system in comparison to the distal roots. The median of major diameters of mesiobuccal, mesiolingual and single mesial canals were: 0.34, 0.41 and 0.60 mm, respectively. The higher values of major diameters were found in the distobuccal canals (0.56 mm) and the lower diameters in the distolingual canals (0.29 mm). The lowest orifice distance was found between the mesial canals (MB-ML) and the highest distance between the distal root canals (DB-DL). Almost all distal roots had one root canal and one apical foramen with few accessory canals.

**Conclusions:**

Distolingual root generally has short length, severe curvature and a single root canal with low apical diameter.

## INTRODUCTION

The understanding of the number of canals of human teeth, their transverse section and possible variations, is of utmost importance to achieve the decontamination goals of endodontic therapy because necrotic tissue in untreated root canals can lead to persistent chronic apical periodontitis^4^. Despite the many anatomical variations of the root canal system of the mandibular first molar, the external anatomy typically has two well-defined roots in the majority of cases^24^. The exception to this rule is the occurrence of a supernumerary distolingual root called radix entomolaris. An additional less prevalent variant includes the presence of a root located at the mesiobuccal side denominated radix paramolaris^3^.

As with other anatomical variations including C-shaped mandibular second molars^16^, it has been shown that the incidence of a third root in the mandibular first molar is closely related to ethnicity^8^. This variability has higher prevalence in specific populations, e.g. Mongoloid, Native American, Eskimo and Chinese, for which it can be considered a normal finding^6,11,25^.

Currently, images of cone beam computed tomography (CBCT) have been used to study the prevalence of three-rooted mandibular molars in several populations^23,25^. In addition to the CBCT method, micro-computed tomography (micro-CT) has also been used to describe several morphometric aspects of three-rooted mandibular molars including pulp chamber, curvature and morphometric analysis.

A previous prevalence study in a Brazilian population using CBCT did not find any three-rooted mandibular first molars^20^. Until now, few studies have addressed their prevalence and compared qualitative and quantitative data of the root canal systems of this anatomical variation^11,22^. The purpose of this study was to investigate the prevalence of three-rooted permanent mandibular first molars in a Brazilian population using CBCT images of patients who had undergone CBCT scanning for implant or third molar surgery treatment planning and to analyze *in vitro* the morphometric aspects of the internal anatomy of three-rooted mandibular first molars through micro-CT.

## MATERIAL AND METHODS

### CBCT analysis

Cone beam computed tomography (CBCT) images of mandibular molars were collected from 116 patients who had undergone CBCT scanning for implant or third molar surgery treatment planning. The inclusion criteria were images displaying fully matured and erupted mandibular first molars bilaterally, without root canal fillings, posts or crown restorations. The exclusion criteria were images lacking technical quality or absence of one of the teeth to be evaluated. All the CBCT images were acquired using an i-CAT CBCT device (Imaging Sciences International, Inc, Hatfield, PA, USA). The scanner was operated at 120 kVp, 8 mA and a voxel size of 0.25 mm. Sagittal, coronal and axial images were analyzed with the use of the i-CAT Vision software by an experienced Oral and Maxillofacial radiologist in order to determine the number of roots in the mandibular first molars.

### MicroCT analysis

This study was approved according to the guidelines of the local Human Research Ethics Committee. For the *in vitro* analysis, 55 mandibular first molars with three roots were selected from a pool of extracted teeth. Patients' gender and age were unknown. All teeth were scanned with a micro-CT system (SkyScan 1174v2; Bruker-microCT, Kontich, Belgium) using 50 kV, 800 mA, an isotropic resolution of 19.6 μm, a rotation step of 0.8 degrees and 360-degree rotation. Radiographs of each specimen were reconstructed with dedicated software (NRecon v. 1.6.3; Bruker-microCT, Kontich, Belgium) providing axial cross-sections of the inner structure of the teeth.

### Tridimensional classification

Three-dimensional models were reconstructed after binarization of the source images, exported as P3G files, using the CTAn software (Bruker-microCT, Kontich, Belgium). DataViewer v.1.4.4 (SkyScan, Kontich, Belgium) and CTVol softwares (Bruker-microCT, Kontich, Belgium) were used for visualization and qualitative evaluation of the specimens according to Vertucci's classification^24^. The presence of apical delta, the number of foramina and furcations, the lateral and accessory canals were also recorded. Furthermore, the length of all roots was measured from the junction of the cementoenamel with the apex^13^ using the DataViewer software.

### Morphometric analysis of the cross-sections of the root canal

**Figure 1:**
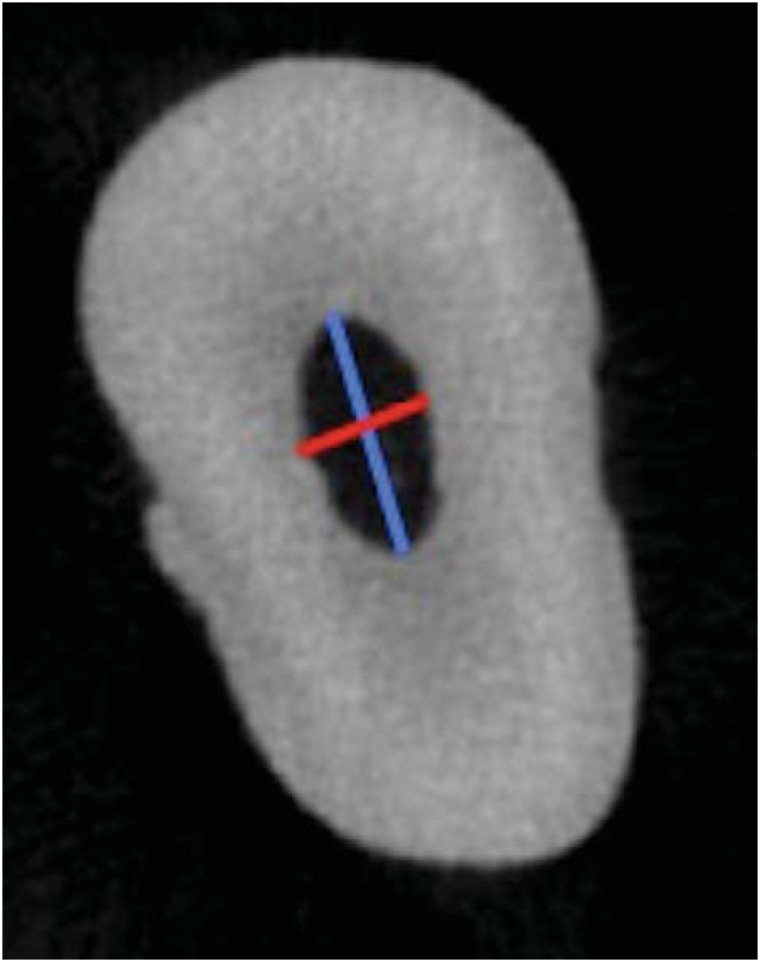
MicroCT cross-section demonstrating the major diameter (blue line) and minor diameter (red line). The major diameter was determined by drawing a line between the two most distant pixels of the root canal walls. The minor diameter was defined as the longest line drawn perpendicular to that of the major diameter^11^

Two-dimensional cross-sections were selected from 1 to 3 mm apical level for quantitative analysis of area, perimeter, roundness, major diameter, minor diameter and aspect ratio using the CTAn software. Area and perimeter were calculated using the Pratt algorithm. The cross-sectional appearance (i.e. round or more ribbon-shaped) was expressed as roundness. The value of roundness ranges from 0-1, with 1 meaning a perfect circle^10^. Major and minor diameters were defined as shown in Figure 1. The aspect ratio is a quantitative index that also helps to describe the shape of the root canal. It is defined as the ratio between major diameter and minor diameter, i.e., the closer the values are to 1 the less flattened the canals are.

### Morphology of the pulp floor

The interorifice distances on the pulp floor were measured using the DataViewer software. A line was drawn between the centers of the orifices and the distances were measured using the geometric measurement module.

### Curvature of the distolingual root

The canal curvature of the distolingual root in the buccolingual direction was measured using the Image J v. 1.46 software (National Institutes of Health, Bethesda, MD, USA) as described by Schneider^18^ (1971) with modifications by Gu, et al.^13^ (2011). It was classified into three groups: straight (10 degrees or less), moderate (10 to 20 degrees) or severe (20 degrees or more). Other anatomical landmarks measured included the distance between pulp chamber floor and beginning of the curvature and from this point to the apical foramen.

### Statistical analysis

The results of the 2D analysis, the angles and the distances between the anatomic landmarks were described as having median, minimum and maximum values. The analysis of the interorifice distances on the pulp floor and the morphometric analysis of the cross-sections of the root canal did not show normal distributions thus nonparametric tests were used. Data was statistically compared using Kruskal-Wallis *post-hoc* Dunn test, with the significance level set at p<0.05, using GraphPad Prism 5 (GraphPad Software Inc, La Jolla, CA, USA). The categorization using Vertucci's classification was presented descriptively.

## RESULTS

### CBCT analysis

**Figure 2:**
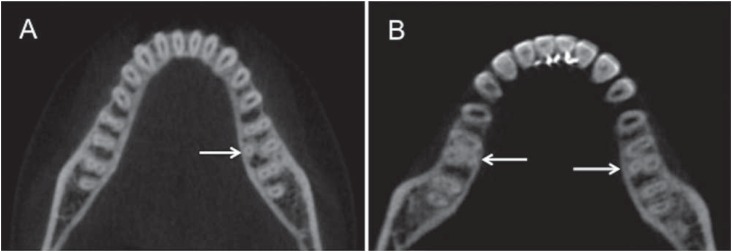
Cone-beam computed tomography images: (A) mandibular first molar with a distolingual root; (B) bilateral mandibular first molars with distolingual roots

A total of 232 mandibular first molars from a sample of 116 patients were analyzed. Three patients (2 women and 1 man) had three-rooted first molars (2.58%). One bilateral case was observed, therefore, a total of 4 three-rooted mandibular first molars were found, all of which had distolingual root (radix entomolaris) (Figure 2). The radix paramolaris anatomical variation was not present in the mandibular first molars.

### Micro-CT qualitative and quantitative analysis

**Figure 3:**
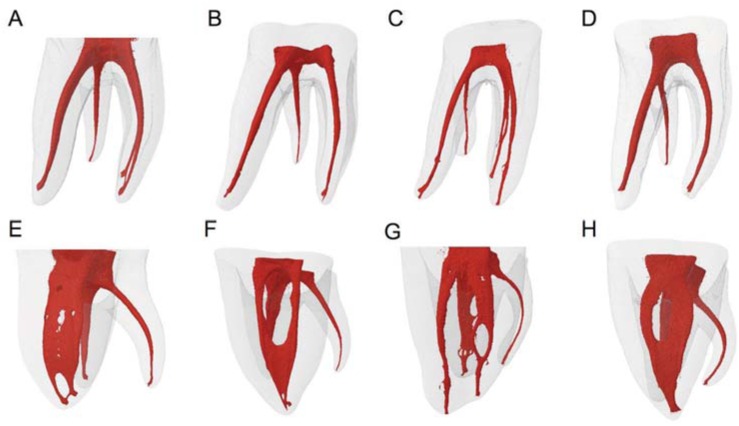
3D reconstructions of three-rooted mandibular first molars observed from the buccal view (A-D). The short length of the distolingual root is observed. The mesial view of these molars (E-H) shows a more complex anatomical root canal system of the mesial root, compared to the distobuccal and distolingual roots and the presence of severe curvatures in the distolingual root

In distolingual roots, the most prevalent canal configuration was Vertucci type I (96.36%) (Figure 3). Only one case had type V anatomy and another, type VII. Type I anatomy was also found more frequently in distobuccal roots (92.72%) followed by type III (one tooth), type VII (one tooth). The mesial root showed a more complex distribution of the root canals: Vertucci type I in 16.36%, type II in 14.55%, type III in 7.27%, type IV in 5.45%, type V in 7.27%, type VI in 10.91% and type VII in 1.82% (Table 1). Root length, presence of apical delta, lateral and accessory canals, and number of foramina, are shown in Table 2. A furcation canal was observed in only one tooth.

**Table 1:** Distribution of Vertucci's Classification types between mesial, distobuccal and distolingual roots

Vertucci's classification	Mesial root	Distobuccal root	Distolingual root
	n (%)	n (%)	n (%)
Type I (1-1 canal)	9 (16.36%)	51 (92.72%)	53 (96.36%)
Type II (2-1 canal)	8 (14.55)%	–	–
Type III (1-2-1 canal)	4 (7.27%)	1 (1.82%)	–
Type IV (2-2 canal)	3 (5.45%)	–	–
Type V (1-2 canal)	4 (7.27%)	–	1 (1.82%)
Type VI (2-1-2 canal)	6 (10.91%)	–	–
Type VII (1-2-1-2 canal)	1 (1.82%)	1 (1.82%)	1(1.82%)
Other types	20 (36.36%)	2 (3.64%)	–

**Table 2:** Characteristics of mandibular first molars observed on micro-CT: Median root length (minimum - maximum). Significant statistical differences were observed between all groups of roots (ANOVA, Turkey's Multiple Comparision Test). Configuration of root canal system showing the prevalence of apical delta, lateral and accessory canals. Distribution of number of foramina for each group

Column1	Mesial	Distobuccal	Distolingual
Root length (mm)	14.02	12.58	11.55
	(10.41-17.50)	(8.51-15.40)	(7.84-16.11)
Apical delta	8	7	6
Lateral canals	4	3	1
Accessory canals	1	3	2
1 foramen	32.7%	84%	80%
2 foramina	50%	14%	20%
3 foramina	17.3%	2%	0

At 1 mm apical level, the lowest area values were found in mesiobuccal, mesiolingual and distolingual root canals (p>0.05). The highest values for area and perimeter parameters were found in the distobuccal and single mesial canals (Table 3).

**Table 3:** Morphometric parameters of the evaluated roots at 1, 2 and 3 mm apical level MB (mesiobuccal), ML (mesiolingual), DB (distobuccal), DL (distolingual), M (single mesial)

	MB	ML	DB	DL	M single
	Median (Minimum-Maximum)	Median (Minimum-Maximum)	Median (Minimum-Maximum)	Median (Minimum-Maximum)	Median (Minimum-Maximum)
1 mm apical					
Area (mm^2^)	0.05 (0.01-0.80)^a^	0.07 (0.01-0.79)^a^	0.13 (0.04-0.72)^b^	0.04 (0.01-0.54)^a^	0.13 (0.05-1.35)^b^
Perimeter (mm)	0.95 (0.46-5.63)^a^	1.16 (0.34-5.56)^a^	1.48 (0.83-3.82)^b^	0.78 (0.30-3.23)^a^	1.61 (0.24-5.34)^b^
Roundness	0.69 (0.16-0.85)^ab^	0.59 (0.17-0.78)^a^	0.56 (0.22-0.80)^a^	0.69 (0.34-0.91)^b^	0.47 (0.22-0.80)^a^
Major diameter (mm)	0.34 (0.16-2.50)^ab^	0.41 (0.12-2.40)^b^	0.56 (0.29-1.51)^b^	0.29 (0.11-1.22)^a^	0.60 (0.15-1.88)^b^
Minor diameter (mm)	0.23 (0.05-0.82)^a^	0.23 (0.03-0.54)^a^	0.35 (0.15-0.74)^b^	0.19 (0.07-0.59)^a^	0.33 (0.15-3.78)^b^
Aspect Ratio	1.36 (0.92-5.76)^ab^	1.55 (1.06-4.62)^ab^	1.55 (0.0-3.53)^ab^	1.36 (0.97-3.18)^a^	2.05 (1.23-4.17)^b^
2 mm apical					
Area (mm^2^)	0.11 (0.03-0.85)^a^	0.10 (0.02-0.98)^ab^	0.17 (0.05-0.68)^ab^	0.06 (0.01-0.75)^a^	0.19 (0.08-0.86)^b^
Perimeter (mm)	1.44 (0.72-6.62)^b^	1.45 (0.61-7.38)^bc^	1.61 (0.84-4.29)^bc^	0.92 (0.26-3.47)^a^	2.25 (1.18-4.57)^c^
Roundness	0.52 (0.08-0.85)^ab^	0.39 (0.08-0.80)^a^	0.59 (0.16-0.91)^b^	0.72 (0.13-0.87)^c^	0.30 (0.10-0.55)^a^
Major diameter (mm)	0.50 (0.24-2.92)^b^	0.57 (0.23-3.24)^b^	0.58 (0.27-1.82)^bc^	0.33 (0.09-1.29)^a^	0.99 (0.44-1.83)^c^
Minor diameter (mm)	0.27 (0.13-0.86)^a^	0.25 (0.13-0.63)^a^	0.37 (0.15-0.90)^b^	0.25 (0.03-0.74)^ab^	0.33 (0.19-0.96)^ab^
Aspect Ratio	1.84 (0.92-9.58)^bc^	2.21 (1.13-9.58)^c^	1.59 (0.99-5.75)^ab^	1.31 (0.91-4.34)^a^	3.03 (1.47-7.21)^c^
3 mm apical					
Area (mm^2^)	0.17 (0.04-1.25)^b^	0.16 (0.03-1.30)^b^	0.25 (0.07-0.80)^bc^	0.07 (0.01-0.92)^a^	0.27 (0.17-1.45)^c^
Perimeter (mm)	1.72 (0.78-8.02)^b^	2.02 (0.72-7.49)^b^	2.18 (1.04-5.36)^bc^	1.05 (0.35-3.70)^a^	3.49 (1.95-7.27)^c^
Roundness	0.37 (0.06-0.80)^ab^	0.44 (0.07-0.76)^a^	0.60 (0.14-0.87)^b^	0.75 (0.39-0.89)^c^	0.20 (0.05-0.43)^a^
Major diameter (mm)	0.68 (0.26-3.57)^b^	0.69 (0.29-3.29)^b^	0.79 (0.34-1.71)^b^	0.36 (0.12-1.31)^a^	1.54 (0.82-2.97)^c^
Minor diameter (mm)	0.33 (0.10-0.86)^a^	0.31 (0.15-0.66)^a^	0.46 (0.19-0.94)^b^	0.31 (0.11-0.90)^a^	0.31 (0.19-0.74)^ab^
Aspect Ratio	2.29 (1.13-9.71)^c^	2.25 (1.15-9.71)^c^	1.55 (1.04-4.54)^b^	1.26 (0.92-2.51)^a^	4.77 (2.21-10.06)^c^

Different superescript letters (a, b and c) in the same row indicate statistical difference between the groups (p<0.05).

Distolingual canals showed higher roundness values and lower aspect ratio values in comparison to the other root canals evaluated (p<0.05). The median of major diameters of mesiobuccal and mesiolingual and single mesial canals were as follows: 0.34, 0.41 and 0.60 mm, respectively. The highest values of major diameters were found in the distobuccal canals (0.56 mm) and the lowest values in the distolingual canals (0.29 mm). Other values corresponding to 2 and 3 mm apical levels are shown in Table 3.

**Figure 4:**
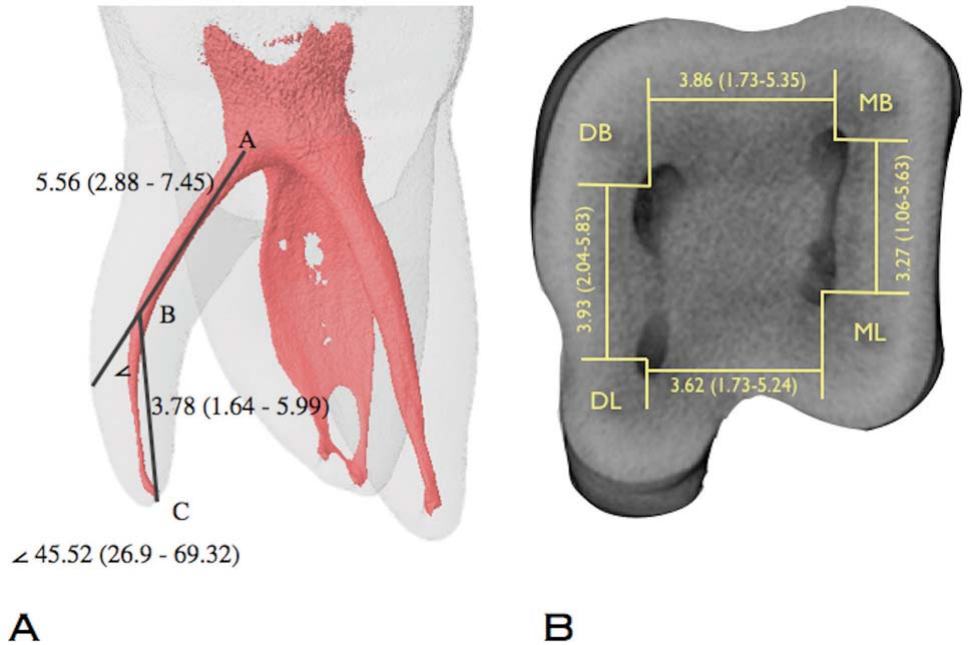
(A) The median and range values of the distance between the pulp chamber floor and the beginning of the curvature (A-B) and the distance between the curvature and the apical foramen (B-C) in the distolingual root. (<) The median and range values of the angle of curvature in the distolingual root. The median and range values of the interorifice distances at 1 mm from the pulp chamber floor can be observed in (B)

All the distolingual roots exhibited severe curvatures (100%) Figure 4A. The shortest orifice distance was found between the mesial canals (MB-ML) and the longest distance between the distal root canals (DB-DL) (p<0.05) Fig. 4B.

## DISCUSSION

The identification of additional canal systems in the mesial and distal root of mandibular molars is considered important for successful disinfection and filling of the root canal system, and consequently for the long-term prognosis of the endodontically treated tooth. The presence of a third root, usually a distolingual root, is the most common anatomical variation in mandibular molars^7^.

It seems plausible that ethnical background is a major factor that influences the prevalence of a distolingual root, as previous studies have demonstrated higher prevalence among individuals of Asian origin, varying from 24.5% to 33.3%^6,21,23,25,26^. The prevalence found in our study is similar to what was reported by Shemesh, et al.^19^ (2015), in an Israeli population with CBCT.

Ferraz & Pecora^9^ (1992) observed a similar prevalence of three-rooted mandibular molars in a Brazilian population (2.8% for Black origin and 4.2% for Caucasian). However, this study was carried out with periapical radiographs. In a CBCT study with a Brazilian population, Silva, et al.^20^ (2013) did not find any three-rooted first molars.

To identify an additional root in mandibular molars, changes in the horizontal angulation during X-ray exposure may be useful, in order to attempt to overcome the limitations of radiographs, such as superimpositions by surrounding structures. However, the addition of multiple radiographs does not ensure the identification of this anatomical variability^1-22^. Due to this fact, although CBCT should not be used as a routine procedure in endodontics, it may be indicated in the assessment and treatment of complex endodontic conditions^2^, because this technique provides a better visualization of anatomical variations in the number of roots and root canals^1,22^. According to Abella, et al.^1^ (2011), when an additional root is detected before root canal treatment, the clinician can plan the procedures better, such as enlarging the opening access cavity in order to locate all the orifices of the canals.

In the present study, 55 three-rooted mandibular first molars were evaluated through micro-CT analysis, a number that can be considered superior to previous anatomical studies which addressed the morphometric aspects of this variation through micro-CT^11-13,22^.

Almost all distolingual and distobuccal roots had one root canal (Vertucci type I) and one apical foramen. The distobuccal and distolingual roots had a single canal in 92.72% and 96.36% of the cases, respectively, which contrasts with the lower prevalence of single canals of distal roots of two-rooted mandibular first molars (60-71%)^10,14^. On the contrary, the mesial root showed a more complex distribution of the internal anatomy with the presence of two foramina being the most common finding (50%). Among mesial roots, single canals were markedly less common (16.36%) and a large variability in Vertucci's classification was observed. In this study, the distal roots results are similar to the categories found by Gu, et al.^11^ (2010) and Wang, et al.^25^ (2010). On the other hand, mesial roots showed a different distribution which contrasts with the work of these authors. The description of the mesial and distal roots of mandibular molars according to Vertucci's classification and micro-CT imaging has been restricted to only one previous study, which observed 81.8% of distal roots with a single canal^15^. One can thus speculate that the complex anatomy of distal roots is less frequent when an extra distolingual root is present.

Gu, et al.^11^ (2010) found out that accessory and lateral canals rarely occurred in distolingual roots. Similarly, in this study, lateral and accessory canals were found in only one and two cases, respectively. However, such anatomical variations are also not common in mesial and distobuccal roots.

The analysis of morphometric data at 1 mm level demonstrated lower median values of apical diameter in the distolingual canals (0.30 mm) compared to distobuccal canals (0.56 mm). In addition, distolingual canals are rounder in shape, whereas the mesial and distobuccal canals are significantly more oval shaped, which is in agreement with the studies by Gu, et al.^13^ (2011) and Souza-Flamini, et al.^22^ (2014). In this investigation, the major apical diameter values were similar to the ones previously reported by Harris, et al.^15^ (2013).

According to the present study, the distance of the orifices on the pulpal floor suggests that the endodontic access should be enlarged from a triangular to a trapezoidal opening with an extension to the distolingual area to help locating the DL canal^3,7^. The use of an operative microscope can be useful in detecting a third root in a mandibular molar. Once the additional root is identified in the preoperative diagnosis radiograph or CBCT, the distance between the mesiobuccal and mesiolingual roots can be used as a guide for finding the distolingual root canal since the distance between the distobuccal and distolingual roots is usually between 0.5 and 1 mm longer than the mesiobuccal to mesiolingual distance^11^.

Studies have shown that the additional distolingual root is generally smaller than the mesial and distobuccal roots^5,13,22^ and has severe curvature^12^. In a previous study^13^, the mean length of the distolingual root was 10.65 mm with a curvature of 32 degrees, which is similar to the results of the present study. Another study in a Brazilian population showed a lower mean length i.e. 7.65 mm^22^. The variation can be explained by the difference in the number of samples studied, since Souza-Flamini, et al.^22^ (2014) used only 19 teeth.

Clinicians need to know about the short length and severe curvature of distolingual roots because it can increase the risk of accidents such as instrument separation or ledge formation. It is known that cyclic fatigue decreases with an increase in the angle of curvature^17^. Thus, decreasing taper conicity, using smaller apical diameters, nickel-titanium files and preflaring of the cervical third are indicated in order to avoid accidents.

Considering the prevalence and characteristics of the distolingual root, clinicians should be able to diagnose and develop skills to provide adequate root canal treatment when this variation is present.

## CONCLUSION

The prevalence of three-rooted mandibular molars in a Brazilian subpopulation was of 2.58%. Distolingual roots had short length, severe curvature and a low apical diameter in comparison to the distobuccal and mesial roots. Single canals were highly prevalent in both distal roots in comparison to the mesial root which showed a more complex anatomical distribution.
